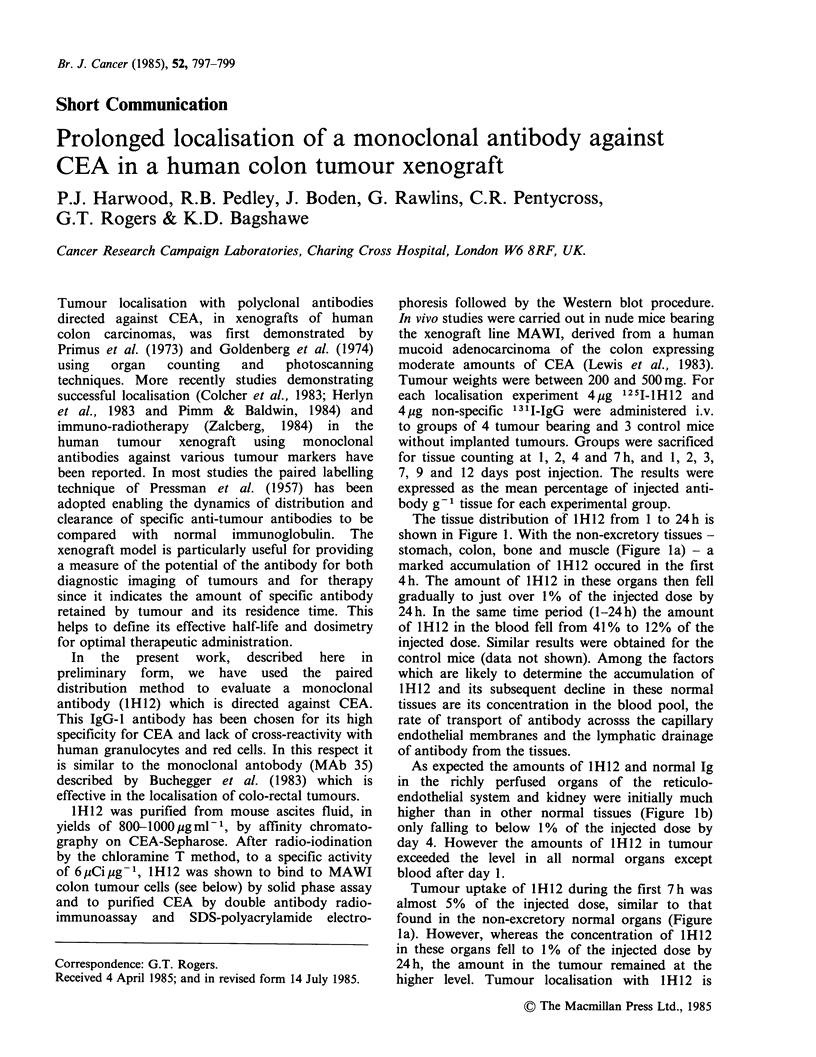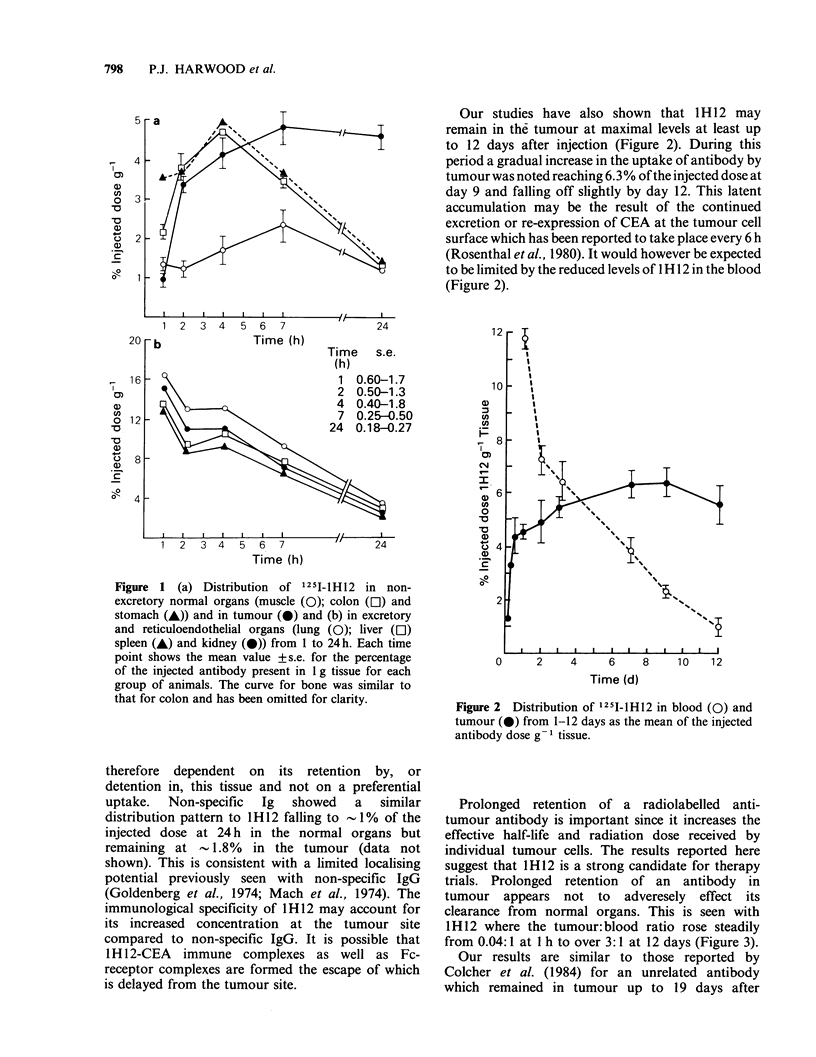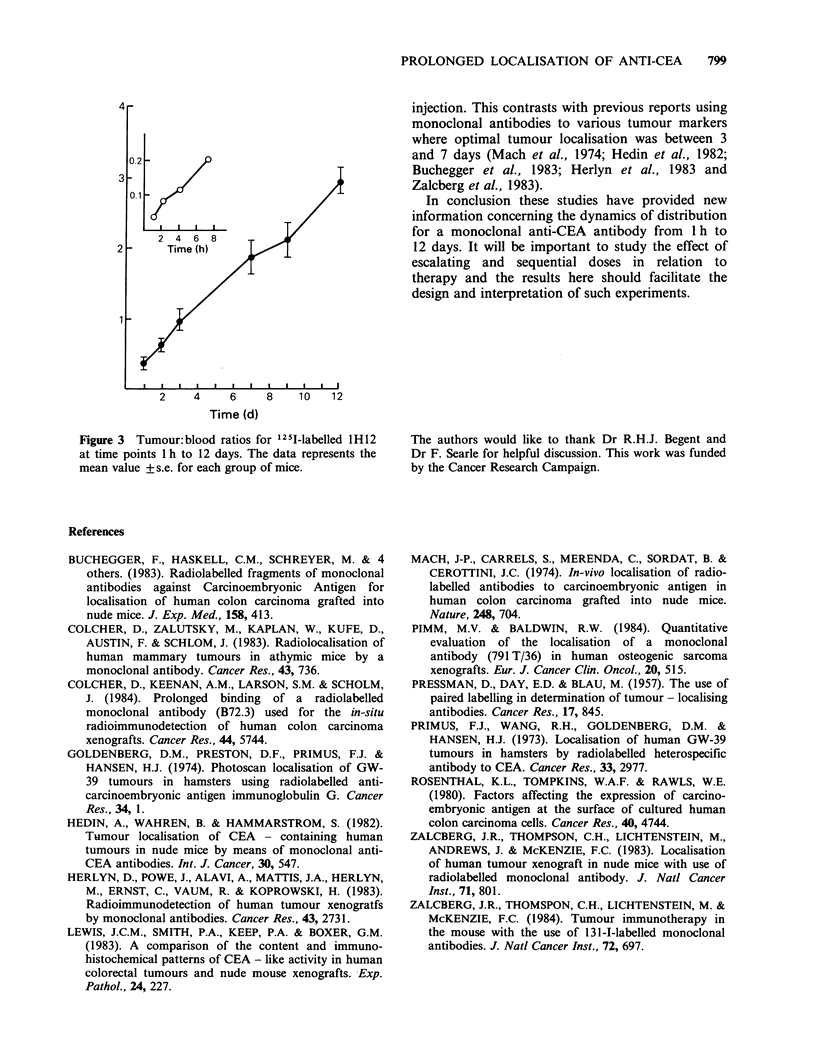# Prolonged localisation of a monoclonal antibody against CEA in a human colon tumour xenograft.

**DOI:** 10.1038/bjc.1985.261

**Published:** 1985-11

**Authors:** P. J. Harwood, R. B. Pedley, J. Boden, G. Rawlins, C. R. Pentycross, G. T. Rogers, K. D. Bagshawe


Br. J. Cancer (1985), 52, 797–799

### Short Communication

### Prolonged localisation of a monoclonal antibody against CEA in a human colon tumour xenograft

P.J. Harwood, R.B. Pedley, J. Boden, G. Rawlins, C.R. Pentycross, G.T. Rogers & K.D. Bagshawe

*Cancer Research Campaign Laboratories, Charing Cross Hospital, London W6 8RF, UK.*

Tumour localisation with polyclonal antibodies directed against CEA, in xenografts of human colon carcinomas, was first demonstrated by Primus *et al.* (1973) and Goldenberg *et al.* (1974) using organ counting and photoscanning techniques. More recently studies demonstrating successful localisation (Colcher *et al.*, 1983; Herlyn *et al.*, 1983 and Pimm & Baldwin, 1984) and immuno-radiotherapy (Zalcberg, 1984) in the human tumour xenograft using monoclonal antibodies against various tumour markers have been reported. In most studies the paired labelling technique of Pressman *et al.* (1957) has been adopted enabling the dynamics of distribution and clearance of specific anti-tumour antibodies to be compared with normal immunoglobulin. The xenograft model is particularly useful for providing a measure of the potential of the antibody for both diagnostic imaging of tumours and for therapy since it indicates the amount of specific antibody retained by tumour and its residence time. This helps to define its effective half-life and dosimetry for optimal therapeutic administration.

In the present work, described here in preliminary form, we have used the paired distribution method to evaluate a monoclonal antibody (1H12) which is directed against CEA. This IgG-1 antibody has been chosen for its high specificity for CEA and lack of cross-reactivity with human granulocytes and red cells. In this respect it is similar to the monoclonal antobody (MAb 35) described by Buchegger *et al.* (1983) which is effective in the localisation of colo-rectal tumours.

1H12 was purified from mouse ascites fluid, in yields of 800–1000 $\mu g\,ml^{-1}$, by affinity chromatography on CEA-Sepharose. After radio-iodination by the chloramine T method, to a specific activity of 6 $\mu Ci\,\mu g^{-1}$, 1H12 was shown to bind to MAWI colon tumour cells (see below) by solid phase assay and to purified CEA by double antibody radioimmunoassay and SDS-polyacrylamide electrophoresis followed by the Western blot procedure. *In vivo* studies were carried out in nude mice bearing the xenograft line MAWI, derived from a human mucoid adenocarcinoma of the colon expressing moderate amounts of CEA (Lewis *et al.*, 1983). Tumour weights were between 200 and 500 mg. For each localisation experiment 4 $\mu g$ $^{125}I$-1H12 and 4 $\mu g$ non-specific $^{131}I$-IgG were administered i.v. to groups of 4 tumour bearing and 3 control mice without implanted tumours. Groups were sacrificed for tissue counting at 1, 2, 4 and 7 h, and 1, 2, 3, 7, 9 and 12 days post injection. The results were expressed as the mean percentage of injected antibody $g^{-1}$ tissue for each experimental group.

The tissue distribution of 1H12 from 1 to 24 h is shown in Figure 1. With the non-excretory tissues – stomach, colon, bone and muscle (Figure 1a) – a marked accumulation of 1H12 occured in the first 4 h. The amount of 1H12 in these organs then fell gradually to just over 1% of the injected dose by 24 h. In the same time period (1–24 h) the amount of 1H12 in the blood fell from 41% to 12% of the injected dose. Similar results were obtained for the control mice (data not shown). Among the factors which are likely to determine the accumulation of 1H12 and its subsequent decline in these normal tissues are its concentration in the blood pool, the rate of transport of antibody acrosss the capillary endothelial membranes and the lymphatic drainage of antibody from the tissues.

As expected the amounts of 1H12 and normal Ig in the richly perfused organs of the reticulo-endothelial system and kidney were initially much higher than in other normal tissues (Figure 1b) only falling to below 1% of the injected dose by day 4. However the amounts of 1H12 in tumour exceeded the level in all normal organs except blood after day 1.

Tumour uptake of 1H12 during the first 7 h was almost 5% of the injected dose, similar to that found in the non-excretory normal organs (Figure 1a). However, whereas the concentration of 1H12 in these organs fell to 1% of the injected dose by 24 h, the amount in the tumour remained at the higher level. Tumour localisation with 1H12 is

Correspondence: G.T. Rogers.
Received 4 April 1985; and in revised form 14 July 1985.

© The Macmillan Press Ltd., 1985

**Figure 1** (a) Distribution of $^{125}$I-1H12 in non-excretory normal organs (muscle (○); colon (□) and stomach (▲)) and in tumour (●) and (b) in excretory and reticuloendothelial organs (lung (○); liver (□) spleen (▲) and kidney (●)) from 1 to 24 h. Each time point shows the mean value ±s.e. for the percentage of the injected antibody present in 1 g tissue for each group of animals. The curve for bone was similar to that for colon and has been omitted for clarity.

therefore dependent on its retention by, or detention in, this tissue and not on a preferential uptake. Non-specific Ig showed a similar distribution pattern to 1H12 falling to ~1% of the injected dose at 24 h in the normal organs but remaining at ~1.8% in the tumour (data not shown). This is consistent with a limited localising potential previously seen with non-specific IgG (Goldenberg *et al.*, 1974; Mach *et al.*, 1974). The immunological specificity of 1H12 may account for its increased concentration at the tumour site compared to non-specific IgG. It is possible that 1H12-CEA immune complexes as well as Fc-receptor complexes are formed the escape of which is delayed from the tumour site.

Our studies have also shown that 1H12 may remain in the tumour at maximal levels at least up to 12 days after injection (Figure 2). During this period a gradual increase in the uptake of antibody by tumour was noted reaching 6.3% of the injected dose at day 9 and falling off slightly by day 12. This latent accumulation may be the result of the continued excretion or re-expression of CEA at the tumour cell surface which has been reported to take place every 6 h (Rosenthal *et al.*, 1980). It would however be expected to be limited by the reduced levels of 1H12 in the blood (Figure 2).

**Figure 2** Distribution of $^{125}$I-1H12 in blood (○) and tumour (●) from 1–12 days as the mean of the injected antibody dose g$^{-1}$ tissue.

Prolonged retention of a radiolabelled anti-tumour antibody is important since it increases the effective half-life and radiation dose received by individual tumour cells. The results reported here suggest that 1H12 is a strong candidate for therapy trials. Prolonged retention of an antibody in tumour appears not to adveresely effect its clearance from normal organs. This is seen with 1H12 where the tumour:blood ratio rose steadily from 0.04:1 at 1 h to over 3:1 at 12 days (Figure 3).

Our results are similar to those reported by Colcher *et al.* (1984) for an unrelated antibody which remained in tumour up to 19 days after

**Figure 3** Tumour:blood ratios for $^{125}$I-labelled 1H12 at time points 1 h to 12 days. The data represents the mean value ±s.e. for each group of mice.

injection. This contrasts with previous reports using monoclonal antibodies to various tumour markers where optimal tumour localisation was between 3 and 7 days (Mach *et al.*, 1974; Hedin *et al.*, 1982; Buchegger *et al.*, 1983; Herlyn *et al.*, 1983 and Zalcberg *et al.*, 1983).

In conclusion these studies have provided new information concerning the dynamics of distribution for a monoclonal anti-CEA antibody from 1 h to 12 days. It will be important to study the effect of escalating and sequential doses in relation to therapy and the results here should facilitate the design and interpretation of such experiments.

The authors would like to thank Dr R.H.J. Begent and Dr F. Searle for helpful discussion. This work was funded by the Cancer Research Campaign.

### References

BUCHEGGER, F., HASKELL, C.M., SCHREYER, M. & 4 others. (1983). Radiolabelled fragments of monoclonal antibodies against Carcinoembryonic Antigen for localisation of human colon carcinoma grafted into nude mice. *J. Exp. Med.*, **158**, 413.

COLCHER, D., ZALUTSKY, M., KAPLAN, W., KUFE, D., AUSTIN, F. & SCHLOM, J. (1983). Radiolocalisation of human mammary tumours in athymic mice by a monoclonal antibody. *Cancer Res.*, **43**, 736.

COLCHER, D., KEENAN, A.M., LARSON, S.M. & SCHOLM, J. (1984). Prolonged binding of a radiolabelled monoclonal antibody (B72.3) used for the *in-situ* radioimmunodetection of human colon carcinoma xenografts. *Cancer Res.*, **44**, 5744.

GOLDENBERG, D.M., PRESTON, D.F., PRIMUS, F.J. & HANSEN, H.J. (1974). Photoscan localisation of GW-39 tumours in hamsters using radiolabelled anti-carcinoembryonic antigen immunoglobulin G. *Cancer Res.*, **34**, 1.

HEDIN, A., WAHREN, B. & HAMMARSTROM, S. (1982). Tumour localisation of CEA – containing human tumours in nude mice by means of monoclonal anti-CEA antibodies. *Int. J. Cancer*, **30**, 547.

HERLYN, D., POWE, J., ALAVI, A., MATTIS, J.A., HERLYN, M., ERNST, C., VAUM, R. & KOPROWSKI, H. (1983). Radioimmunodetection of human tumour xenogratfs by monoclonal antibodies. *Cancer Res.*, **43**, 2731.

LEWIS, J.C.M., SMITH, P.A., KEEP, P.A. & BOXER, G.M. (1983). A comparison of the content and immuno-histochemical patterns of CEA – like activity in human colorectal tumours and nude mouse xenografts. *Exp. Pathol.*, **24**, 227.

MACH, J-P., CARRELS, S., MERENDA, C., SORDAT, B. & CEROTTINI, J.C. (1974). *In-vivo* localisation of radio-labelled antibodies to carcinoembryonic antigen in human colon carcinoma grafted into nude mice. *Nature*, **248**, 704.

PIMM, M.V. & BALDWIN, R.W. (1984). Quantitative evaluation of the localisation of a monoclonal antibody (791T/36) in human osteogenic sarcoma xenografts. *Eur. J. Cancer Clin. Oncol.*, **20**, 515.

PRESSMAN, D., DAY, E.D. & BLAU, M. (1957). The use of paired labelling in determination of tumour – localising antibodies. *Cancer Res.*, **17**, 845.

PRIMUS, F.J., WANG, R.H., GOLDENBERG, D.M. & HANSEN, H.J. (1973). Localisation of human GW-39 tumours in hamsters by radiolabelled heterospecific antibody to CEA. *Cancer Res.*, **33**, 2977.

ROSENTHAL, K.L., TOMPKINS, W.A.F. & RAWLS, W.E. (1980). Factors affecting the expression of carcino-embryonic antigen at the surface of cultured human colon carcinoma cells. *Cancer Res.*, **40**, 4744.

ZALCBERG, J.R., THOMPSON, C.H., LICHTENSTEIN, M., ANDREWS, J. & McKENZIE, F.C. (1983). Localisation of human tumour xenograft in nude mice with use of radiolabelled monoclonal antibody. *J. Natl Cancer Inst.*, **71**, 801.

ZALCBERG, J.R., THOMSPON, C.H., LICHTENSTEIN, M. & McKENZIE, F.C. (1984). Tumour immunotherapy in the mouse with the use of 131-I-labelled monoclonal antibodies. *J. Natl Cancer Inst.*, **72**, 697.